# Braving the dark: mental health challenges and academic performance of Ukrainian university students during the war

**DOI:** 10.1007/s00127-025-02867-7

**Published:** 2025-03-03

**Authors:** Irina Pinchuk, Inna Feldman, Violetta Seleznova, Volodymyr Virchenko

**Affiliations:** 1 Institute of Psychiatry, Taras Shevchenko National University of Kyiv, Kyiv, Ukraine; 2 Department of Public Health and Caring Sciences, Uppsala University, Uppsala, Sweden; 3 Faculty of Economics, Taras Shevchenko National University of Kyiv, Kyiv, Ukraine

**Keywords:** Mental health, Mental conditions, War, Academic performance, University students

## Abstract

**Purpose:**

The paper aims to investigate the association of mental health problems with academic performance of university students using data from a cross-sectional survey of Ukrainian university students during the war. The prevalence of mental health problems among students with their subsequent division into different severity groups is investigated.

**Methods:**

The study combines a cross-sectional survey method to collect data and a regression analysis technique to identify mental health problems that negatively associated with students’ academic performance during the war. The survey questionnaire includes a demographic section, mental health screening tools, as well as Work Productivity and Activity Impairment: Special Health Problems (WPAI: SHP) section, adapted for the purpose of the study. The data sample includes responses from 1398 university students from different regions of Ukraine.

**Results:**

According to survey data 85.8% of all respondents had depression symptoms, 66.1%– anxiety symptoms, 56.9%–sleep problems, and 48.1%– PTSD symptoms. Results of regression modeling confirms the devastating effect of mental health problems on academic performance of university students during the war, in particular, a severe depression symptoms, anxiety symptoms and sleep problems are associated with 17.4%, 12.2% and 11.0% decrease in academic performance of university students, respectively.

**Conclusion:**

The prevalence of mental health problems and related academic performance impairment among students during wartime become a challenge for the successful recovery of Ukrainian society and therefore require a quick response at both the institutional and public policy levels.

## Introduction

On February 24, 2022, Russia started an unprovoked full-scale war against Ukraine. It was preceded by an eight-year latent conflict, resulting in tens of thousands of civilian casualties and more than a million internally displaced families [1, 2]. The full-scale Russian invasion has led to unprecedented consequences. Among them are occupation of 18% of the Ukrainian territory, and launching more than 12.5 thousand missiles and attack drones over Ukraine [3, 4]. Direct infrastructural damage is valued at 155 billion dollars, while more than 250 thousand residential buildings were damaged or destroyed [5].

Russian military aggression has severely damaged the Ukrainian education sector, crucial for accumulation of intellectual and human capital for the post-war recovery. Current university students will be the driving force of social and economic activity in Ukraine after the war, so the burden of post-war reconstruction will mostly fall on this population.

Since the beginning of the full-scale war, approximately 3,800 educational institutions have suffered from shelling and bombing, 365 of them were completely destroyed [6]. Students from frontline regions have experienced more than 5,407 h of air raids since February 2022, which is equivalent to 7.5 months of life [7]. Post-war recovery requires significant efforts and resources, and the academic performance of university students is one of the key elements of this process. Minimizing mental health burden among students and ensuring a high level of motivation for studying is crucial to acquire the necessary knowledge and skills for the development of various economic sectors, as well as for maintaining social stability and democratic institutions.

The negative impact of mental health problems on academic performance is a well-known phenomenon adressed in the scientific literature of sociology, epidemiology and social psychiatry [8–10]. Regardless of military conflicts, students are a vulnerable segment in matters of mental health: academic performance and pressure to succeed are among the most common stressors for them [11]. According to Mason [12], college students are much more prone to suicidal thoughts compared to non-students of the same age. Some studies indicate that the early years of education are one of the main risk factors for the development of mental disorders among university students [13]. Mental disorders reduce the chances of enrollment, worsen academic performance, increase the risks of not completing studies, and diminish prospects of favorable employment in the future [14, 15].

War exacerbates the mental health of young people, reducing cognitive abilities and thereby contributing to underachievement. The majority of published studies reported the negative consequencies of war on school children. According to a recent study [16], the armed conflict in Colombia negatively impacted state test results in secondary schools both in the short and long term. Another study indicates a decline in education levels in Iraq due to military actions, reflected in decreased enrollment rates [17]. The war in Syria also profoundly affected student behavior and performance, including an increase in school dropouts, involvement in armed forces, and neglect of educational pursuits [18]. However, little is known about the impact of war trauma on the academic performance of university students.

Three months after the start of the full-scale war, about a third of Ukrainian university students reported symptoms of post-traumatic stress disorder and depression, while the prevalence of anxiety disorder symptoms was over 80% [19]. Accordingly, mental health challenges, which are actively spreading among Ukrainian students during the war, cause an academic-related productivity loss, and this forces students to spend more time to get a satisfactory result in terms of knowledge, grades and competencies. Therefore, the mental health problems among students during wartime goes beyond the healthcare context.

The aim of this study is to investigate the association of mental health problems with academic performance of university students during the war in Ukraine. We believe that the results will help to develope interventions and policies that will contribute to the management of the negative effects of the war and improve the conditions for young people to reconcile their creative and social lives.

## Methods

The study used cross-sectional design and utilized data collected from a large-scale survey for Ukrainian university students (Bachelor’s, Master’s and PhD programs), from different regions of Ukraine conducted at the end of 2023. Data was obtained through online questionnaire using licensed Qualtrics software. The link to the questionnaire was disseminated among departments of education and science, university rectors and the leaders of student government, which then were shared with their students who were encouraged to answer this survey. Data collection was conducted during September– November, 2023. In total, 2,364 students from different regions, excluding the territories temporarily occupied by Russia, anwered the questionnaire in varying degree of completing.

Students were 18 plus years old. All participants were informed that they could revoke their participation at any time, and acquired data would be anonymous and confidential. Regardless of the answers, participants were given guidance on resources they can seek for mental health support. The study was approved by the Ethics Committee of the Institute of Psychiatry of Taras Shevchenko National University of Kyiv (No.1, 17.07.2023), and each of the survey participants signed an informed consent.

The survey included demographic variables, instruments to assess the symptoms of mental problems/disorders, as well as Work Productivity and Activity Impairment: Special Health Problems (WPAI: SHP) Sect. [20], adapted for the purpose of the study. Since students had the option to avoid answering certain questions, the number of students who answered the questions of the WPAI: SHP section was 1,398, therefore the response rate was 59.1% (out of 2,364 students).

Mental health problems were assessed by commonly accepted and evidence based instruments PC-PTSD-5 for post-traumatic stress disorder [21], PHQ-9 to access depression severity [22], GAD-7 for screening and measuring the severity of anxiety [23], ISI for screening sleep problems [24]. The validity and reliability of these scales have been confirmed by numerous systematic reviews that have been published in recent years [25, 26].

In our study, we used version 2.0 of the WPAI: SHP questionnaire, which was developed by Reilly M., Zbrozek A. and Dukes E [20]. The specific feature of this questionnaire is that it allows assessing the impact of any specific health problems on work productivity and activity impairment, in contrast to the original version, which was designed to study the impact of general health problems.

For the purposes of our study, in the WPAI: SHP questionnaire, we replaced the phrase “work productivity” with “academic performance”, “currently employed” with “currently studying at a university”, “from work” with ”from study”, “specific health problems” with “mental health problems”. Additionally, based on experts opinion, we have increased the timeframe from “during the last 7 days” to “during the last 14 days”, as symptoms of some mental disorders usually take a longer time to manifest.

Work Productivity and Activity Impairment: Special Health Problems (WPAI: SHP) questionnaire section included the following six questions: Q1. Are you currently studying at a university? Q2. During the past 14 days, how many hours did you miss from study because of mental health problems? Q3. During the past 14 days, how many hours did you miss from study because of any other reason, such as vacation, holidays, time off to participate in this study? Q4. During the past 14 days, how many hours did you actually study? Q5. During the past 14 days, how much did your mental health problems affect your productivity while you were studying? (use a number between 1 and 10. If your mental health problems have affected your study only a little, choose a low number and vice versa). Q6. During the past 14 days, how much did your mental health problems affect your ability to do your regular daily activities, other than study? (use a number between 1 and 10. If your mental health problems have affected your study only a little, choose a low number and vice versa).

The respondents’ answers to Q5 of the WPAI: SHP section were used to determine the value of the API (Academic Performance Impairment) variable, which could vary from 0 (no negative impact of mental health problems on academic performance) to 10 (maximum negative impact of mental health problems on academic performance). This variable allows us to investigate the association between different mental health problems and students’ academic performance impairment during the war using regression analysis.

In the same way, the respondents’ answers to questions Q2-Q4 were used to construct the HM variable − number of hours that university students missed from study because of mental health problems as a percentage of the total number of hours that university students could spend on studying during the past 14 days. To obtain the value of the HM variable, we divided the respondents’ answer to Q2 by the sum of answers to questions Q2-Q4.

The analysis of the impact of mental health problems on the student’s academic performance was performed in the following steps: (1) combining data on mental health problems into different level of severity groups; (2) testing the reliability of the questionnaire; (3) assessment of the impact of demographic factors and different level of severity of mental health problems separately as independent variables on the API through regression analysis and interpretation of the results.

The data collected was summarized in Microsoft Excel 2019, and then analyzed using the Eviews 12 software. The reliability of the questionnaire was tested using the JASP 0.17.3 software.The impacts of mental health problems/disorders and separate demographic factors on the API were assesed using the linear least squares method in combination with the method of consistent estimation of the McKinnon and White covariance matrix. This approach allowed to take into account the heteroscedasticity of disturbances, as well as to avoid bias and incorrectness of standard estimates of the covariance matrix, which, due to its wide range of applications, holds a key place among the methods of mathematical statistics [27]. This method has been successfully applied in mental health research and allows to assess the patterns observed against the background of random fluctuations of the dependent variable and to use the identified patterns for further forecasts [28].

The scores obtained through respective mesures were used to divide the respondents into groups according to the severity of the symptoms of mental health problems (Table 1).

**Table 1:** Definition of the groups of respondents depending on the severity of symptoms of different mental health problems

Mental health problem	Name of the variable	Severity groups		
		Group I	Group II	Group III
Anxiety	A	No or minimal symptoms: GAD-7 scores from 0 to 4. A = 0	Mild or moderate symptoms: GAD-7 scores from 5 to 14. A = 1	Severe symptoms: GAD-7 scores from 15 to 21. A = 2
Depression	D	No or minimal symptoms: PHQ-9 scores from 0 to 4. D = 0	Mild or moderate symptoms: PHQ-9 scores from 5 to 14. D = 1	Moderately severe or severe symptoms: PHQ-9 scores from 15 to 27. D = 2
PTSD	P	No symptoms: PC-PTSD-5 scores from 0 to 2. *P* = 0	PTSD symptoms: PC-PTSD-5 scores from 3 to 5. *P* = 1	N/A
Sleep disorder	SD	No symptoms: ISI scores from 0 to 7. SD = 0	Mild (sub-threshold) symptoms: ISI scores from 8 to 14. SD = 1	Moderate or severe symptoms: ISI scores from 15 to 28. SD = 2

To analyze the association of different mental health problems with API, we used linear regression, because the available data set was quantitative and met the conditions of normal distribution. The linear regression included independent variables that showed a significant correlation with API as a dependent variable. The independent variables were Age of respondents (AGE), Gender of respondents (GEN; 0– female, 1– male), Year of study of respondents (YEAR), Number of hours that university students missed from study because of mental health problems as a percentage of the total number of hours that university students could spend on studying during the past 14 days (HM), severity of anxiety symptoms (A; 0– minimal/none, 1– mild/moderate, 2 - severe), severity of depression symptoms (D; 0– minimal/none, 1– mild/moderate, 2 - moderately severe/severe), severity of PTSD symptoms (P; 0– none, 1– present), severity of sleep disorder symptoms (SD; 0– none, 1– mild, 2– moderate/severe). To estimate the model parameters by the least square’s method, a sample of answers from 1,398 respondents was used (Table 1).

## Results

### Descriptive statistics

Socio-demographic as well as mental health profiles of Ukrainian university students is presented in Table 2. The 75.5% of respondents were female, which corresponds to the gender distribution in major branches of science within Ukraine’s higher education sector, where women prevail [29]. The median age was 19 years (Mean = 19.55; SD = 3.18). Most of the participants were in the process of getting a Bachelor’s degree (53.3%) and were in the 1st-4th year of study. Only 6.8% of students lived alone, while 82.7% lived with their relatives. The half of the students lived and studied in Ukraine and were not relocated (50.7%). The rest were relocated to other regions of Ukraine (31.2%) or moved abroad (18.1%). The analysis showed that 54.0% of respondents did not seek mental health support, 15.1%– sought professional mental health suppor, while the rest sought support from their relatives or friends (Table 2).

It was predictable that the dynamics of the respondents’ mental health condition and its impact on their study and regular daily activities turned out to be negative. Respondents missed from 2 to 72 class hours due to mental health problems during the last 14 days (Mean = 5.72, SD = 18.74). The median score of API during the last 14 days was 3 (Mean = 3.41, SD = 2.96). Almost the same median score was obtained for the self-rated negative impact of mental health problems on everyday activities during the last 14 days (Mean = 3.43, SD = 2.94).

**Table 2:** Socio-demographic characteristics and mental health conditions of university students during the war in Ukraine

	Socio-demographic characteristics	Categories	Frequency	Percent of respondents
1.	Gender	Female	1,056	75.5%
		Male	342	24.5%
2.	Age	18–20	1,149	82.2%
		21–24	203	14.5%
		25 and more	46	3.3%
3.	Relocation experience	None	709	50.7%
		Another region of Ukraine	436	31.2%
		Abroad	253	18.1%
4.	Living condition	Living with relatives	1,156	82.7%
		Living alone	95	6.8%
		Living with partners or their spouse and children	146	10.4%
		Other	1	0.1%
5.	Year of study	1st Year	293	20.9%
		2nd Year	191	13.7%
		3rd Year	170	12.1%
		4th Year	91	6.5%
		5th Year and higher	90	6.4%
		Not responded	563	40.3%
	**Mental health conditions**	**Mean**	**Median**	**Std. Dev.**
1.	Number of hours that university students missed from study because of mental health problems during the past 14 days	5.72	0	18.74
2.	Number of hours that university students actually study during the past 14 days	34.99	30	25.72
3.	Self-rated negative effect of mental health problems on ability of university students to do their regular daily activities during the past 14 days (scale from 0 to 10)	3.43	3	2.94
	**Academic Performance Impairment (API)**	**Mean**	**Median**	**Std. Dev.**
1.	Self-rated negative effect of mental health problems on academic performance of university students during the past 14 days (scale from 0 to 10)	3.41	3	2.96

**Fig. 1:**
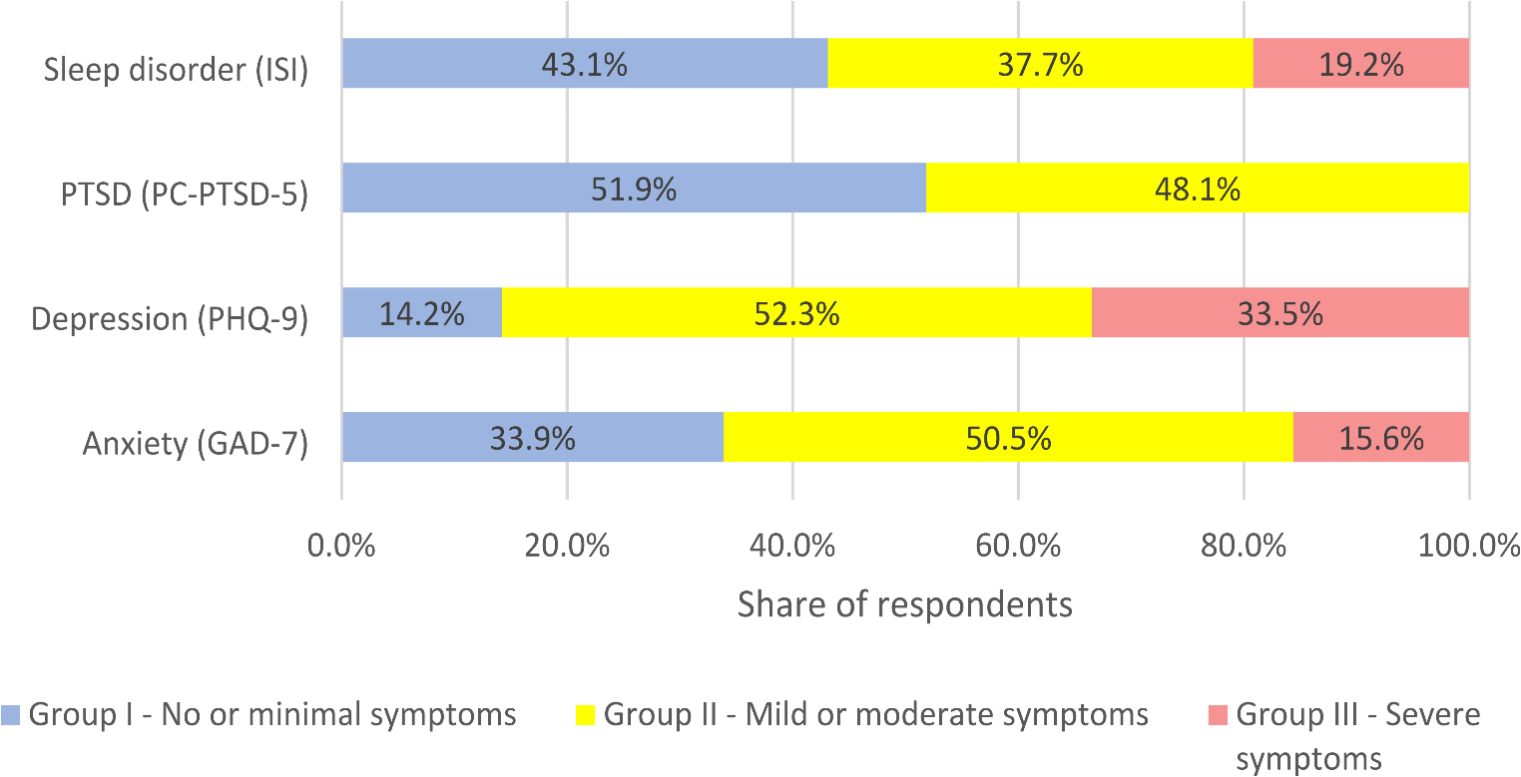
Distribution of mental health problems among Ukrainian university during the war

Regarding mental health problems, 66.1% of all respondents screened anxiety symptoms (mild, moderate and severe) according to the obtained GAD-7 scores, 85.8%– depression symptoms (mild, moderate, moderately severe and severe) according to the obtained PHQ-9 scores, 48.1%– PTSD symptoms according to the obtained PC-PTSD-5 scores, 56.9%– symptoms of sleep disorder (mild, moderate and severe) according to the obtained ISI scores (Fig. 1).

### Reliability of the questionnaire

Before proceeding with the regression analysis, we tested all used questionnaire for reliability using Cronbach’s Alpha and McDonald’s Omega. The results of testing with the JASP 0.17.3 software demonstrated high reliability of the GAD-7 questionnaire (Cronbach’s α = 0.87; McDonald’s ω = 0.88), PHQ-9 questionnaire (Cronbach’s α = 0.85; McDonald’s ω = 0.85), ISI questionnaire (Cronbach’s α = 0.88; McDonald’s ω = 0.88). In contrast, PC-PTSD-5 questionnaire has shown low reliability (Cronbach’s α = 0.58; McDonald’s ω = 0.59).

Reliability testing of the WPAI: SHP questionnaire required preliminary preparation, as questions Q2, Q3, and Q4 are not fully consistent with Q5 and Q6 due to different units and measurement techniques. Questions Q2-Q4 are not scaled and measured in hours that may be specific for different universities, while questions Q5 and Q6 are scaled from 1 to 10. To increase the consistency of the data, we have recalculated the answers to questions Q2, Q3, and Q4 as a percentage of the total amount for Q2-Q4. After that, we conduct reliability test of the WPAI: SHP questionnaire, which showed a fairly good result with Cronbach’s α = 0.73 and McDonald’s ω = 0.98.

### Regression analysis

Originally, we developed a linear regression that included all variables with significant correlation with API. The analysis of the regression parameters demonstrated the problem of multicollinearity and insufficient significance of some independent variables. In particular, the YEAR variable had a significant correlation with the AGE variable and showed an inadequate level of t-statistics. Therefore, it was decided to modify the regression model and exlude variable YEAR. After that, the parameters of the model were estimated by the Least Squares method using the Eviews12 software (Table 3).

**Table 3:** Indicators of the accuracy and reliability of linear regression model

Dependent Variable: API			Method: Least Squares		
Sample: 1,398			Included observations: 1,398		
Variable	Description of variables	Coefficient	Std. Error	t-Statistic	Prob.
C	Intercept	1.976627	0.424105	4.660702	< 0.00001
AGE	Age of respondents	-0.052798	0.020098	-2.627038	0.0087
GEN	Gender of respondents (0– female, 1– male),	-0.405565	0.153068	-2.649567	0.0082
HM	Number of hours that university students missed from study because of mental health problems as a percentage of the total number of hours that university students could spend on studying during the past 14 days	0.047570	0.003906	12.17903	< 0.00001
D	Severity of depression symptoms (0– minimal/none, 1– mild/moderate, 2 - moderately severe/severe)	0.878346	0.130750	6.717727	< 0.00001
A	Severity of anxiety disorder symptoms (0– minimal/none, 1– mild/moderate, 2 - severe),	0.608098	0.126115	4.821762	< 0.00001
SD	Severity of sleep disorder symptoms (0– none, 1– mild, 2– moderate/severe),	0.553477	0.104777	5.282455	< 0.00001
P	PTSD symptoms (0– none, 1– present)	0.380994	0.147355	2.585550	0.0098
R-squared		0.357661	Mean dependent var		3.406295
Adjusted R-squared		0.354426	S.D. dependent var		2.961121
S.E. of regression		2.379190	Akaike info criterion		4.577104
Sum squared resid		7868.160	Schwarz criterion		4.607105
Log likelihood		-3191.395	Hannan-Quinn criter.		4.588320
F-statistic		110.5664	Durbin-Watson stat		1.758770
Prob(F-statistic)		< 0.000001			

To estimate the model parameters by the least square’s method, a sample of answers from 1,398 respondents was used.

The model is presented as a mathematical Eq. (1)

$$\:API\hspace{0.17em}=\hspace{0.17em}1.97\hspace{0.17em}-\hspace{0.17em}0.05*AGE\:-\hspace{0.17em}0.40*GEN\hspace{0.17em}+\hspace{0.17em}0.05*HM\hspace{0.17em}\:$$

1 $$\:+\hspace{0.17em}0.87*D\hspace{0.17em}+\hspace{0.17em}0.61*A\hspace{0.17em}+\hspace{0.17em}0.55*SD\hspace{0.17em}+\hspace{0.17em}0.38*P$$

* description of variables provided in the methodology section and Table 3.

The results of the regression analysis demonstrate the significance and qualitative characteristics of the model. The standard errors of the model are quite small, the F-statistic and all t-statistics are significant, the confidence intervals are appropiate, the Akaike, Schwarz, and Hannan-Quinn criteria have been minimized. The RESET test results are positive and indicate the correct functional form of the model. The analysis of correlation matrices showed the absence of multicollinearity in the model.

The results of the regression modelling proved that mental health problems negatively associated with API of university students during the war in Ukraine. As previously mentioned, the API variable measures the academic performance impairment caused by mental health problems, and therefore a rise in the API value indicates a decrease (i.e., greater impairment) in academic performance of university students (Table 4).

**Table 4:** Interpretation of regression analysis results

Variable	Coefficient	Description of the association between the variable and the API (regressor)
C	1.976627	Value of the regression’s intercept shows the influence of all other factors that were not included in the regression analysis on API
AGE	-0.052798	Older age of the student allows to better coupe with mental health problem and decrease its negative effect on academic performance. In particular, when student becomes 1 year older, the negative effect of mental problems becomes 0.52% lower, which leads to a 0.52% lower API
GEN	-0.405565	Male students (GEN = 1) demonstrated higher resilience to the negative effects of mental health problems, which associated with 4.05% lower reduction in academic performance of male students. Female students (GEN = 0) do not demonstrate any special resilience under deteriorating mental conditions
HM	0.047570	Increase in the share of hours missed from study because of mental health problems (HM) by 1% is negatively associated with a 0.47% decrease in academic performance
D	0.878346	Mild or moderate depression symptoms (D = 1) raise the API value by 0.87 points, which associated with a 8.78% decrease in academic performance of university students. While moderately severe or severe depression symptoms (D = 2) associated with a 17.56% reduction in academic performance of university students
A	0.608098	Mild or moderate anxiety disorder symptoms (A = 1) raise the API value by 0.60 points, which associated with a 6.08% decrease in academic performance of university students. While severe anxiety disorder symptoms (A = 2) associated with a 12.16% reduction in academic performance of university students
SD	0.553477	Mild sleep disorder symptoms (SD = 1) raise the API value by 0.55 points, which associated with a 5.53% decrease in academic performance of university students. While moderate or severe sleep disorder symptoms (SD = 2) associated with a 11.06% reduction in academic performance of university students
P	0.380994	PTSD symptoms (*P* = 1) raise the API value by 0.38 points, which associated with a 3.80% decrease in academic performance of university students.

**Fig. 2:**
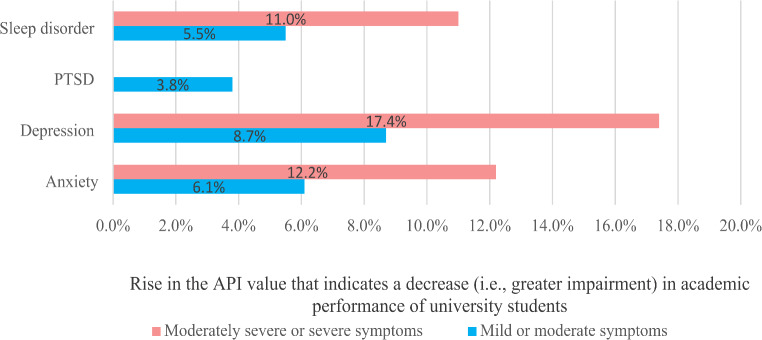
Negative association of different mental health problems with decreasing academic performance of university students during the war in Ukraine

As presented in Eq. (1), mild or moderate depression symptoms raise the API value by 0.87 points, which associated with 8.7% decrease in academic performance of university students. Furthermore, moderately severe or severe depression symptoms associated with 17.4% reduction in academic performance of university students. Regression modelling also confirmed the same negative association of anxiety, PTSD and sleep problems, specifically, severe anxiety disorder symptoms associated with a 12.2% reduction in academic performance, while moderate or severe sleep disorder symptoms associated with a 11% decrease in academic performance of university students. PTSD showed the least negative association with academic performance (3.8% decrease) compared to other mental health problems (Fig. 2).

At the same time, age and gender of the student allows to better coupe with mental health problem and decrease its negative effect on academic performance. In particular, older students are able to better cope with mental health problems, which leads to a 0.5% lower API. Furthermore, male students demonstrated higher resilience to the negative effects of mental health problems, resulting in a 4.0% lower reduction in academic performance of male students. The regression model also confirmed the obvious negative association of student absenteeism with academic performance, as an increase in percentage of hours missed from study because of mental health problems associated with a 0.5% decrease in academic performance.

## Discussion

### Main results

The resuls from this cross-sectional study clearly demonstrate the high prevalence of mental health problems among Ukrainian university students during the war, manifested mainly as moderate and severe symptoms of depression (85.8%) and anxiety (66.1%). Even taking into account the global vulnerability of youth to some mental health problems [30–33], the above mentioned indicators go far beyond the normal context. According to a 2022–2023 web-based survey of United States college students [34], the prevalence was 41% for depression overall and 36% for anxiety disorder. These rates are approximately half of those observed in our study. A survey of French university students conducted in July-August 2021 showed the prevalence of anxiety symptoms at 23.7% and depression at 15.4% [35]. In addition to depression and anxiety, our study highlights an increased share of Ukrainian university students (56.9%) with of sleep problems (mild, moderate and severe).

Our results confirmed that persistance of specific mental health problems negatively associated with reduced academic performance of university students and could created a barrier to the human capital development, which is critical for the postwar recovery of Ukraine. The results of the regression analysis showed that even mild or moderate symptoms of depression, anxiety, PTSD and sleep problems are associated with 8.7%, 6.1%, 3.8% and 5.5% decrease in academic performance of university students, respectively. However, severe symptoms of mental health disorders showed stronger negative association with poor academic performance of university students.

Our study provides an evidence-based finding that the war in Ukraine is not only associated with direct human losses and the destruction of housing and social infrastructure. Using regression analysis, we quantified the hidden negative effect of war on academic performance through symptoms of mental health disorders, which is threatening the future of the nation by reducing the labor potential of young people who cannot acquire the necessary skills during their studies due to the loss of productivity during the war.

### Comparison with previous research

The majority of studies investigated distribution of mental health problems among students are limited in their sample size and survey specific subcategories of students, focusing on particular specialties, years of study, or regions [36–38]. For instance, a cross-sectional study of humanities students in Italy found the prevalence of depressive symptoms to be 30.6% [39], while a survey among undergraduate medical students in the United Arab Emirates reported a nearly 35% prevalence of depression [40]. According to a report on first-year students by the Office for National Statistics in England, 37% of them exhibited moderate or severe depressive symptoms, and 39% showed signs of probable anxiety in various forms [41]. Although the available evidence demonstrates a significantly higher burden of mental health problems among Ukrainian university students compared to student populations in other countries, the fragmented nature of most studies complicates adequate comparisons of mental disorders’ prevalence rates.

Assessing the association of mental health problems with academic performance among Ukrainian students is challenging without proper comparison to other studies on the mental states of populations in war-affected countries. The most of the studies in this area investigates the entire or just the adult population [42–45].

The scientific literature on mental health issues among university students during wartime is very limited and primarily describes the consequences of mental problems acquired against the backdrop of armed conflict for students’ everyday activities, emotional, and physical states. However, war also contributes to the development of mental health problems that hinder students’ academic performance. The association between mental health problems and declining academic performance among school, college, and university students has been thoroughly studied [46–48]. Some publications are devoted to a systematic review of the studies on this topic [48], while others focus on determining the association between mental health problems and academic performance of university students using regression analysis of data collected in the United Arab Emirates [46] and China [49]. Nevertheless, we have not found studies on the impact of mental health problems on academic perfomance that take the war context into account. That is why our study filled an important gap in the literature.

### Strengths and limitations

This study is one of the first to measure the association of war-related mental health problems with the academic performance of university students. Our results based on extentsive dataset collected from representative population of Ukramian students. Furthermore, we used the appropriate instrument to measure specific mental health problems as well as the effect of mental health problems on academic performance and impairment and this allowed us to etsimate the particular contribution of different mental health problems. In this regards, our study opens up prospects for further development in this scientific area. We hope that our findings will draw attention to the necessity of developing strategies and interventions aimed at improving the mental health of students living in wartime conditions. In the medium and long term, this will help preserve the human and intellectual capital needed for post-war recovery.

Despite a rather large sample size of our cross-sectional study, it has several limitations that must be taken into account when interpreting the results of the analysis. First of all, the number of students who answered the questions of the WPAI: SHP section was 1,398, i.e. 59.1% of the survey respondents (2,364 students). Although this section of the questionnaire has an adequate Crombach’s alpha coefficient and the validity of the data is not in doubt, the level of representativeness of the data is lower compared to the total sample of 2,364 survey participants, likely due to the questionnaire’s length, complexity, and disruptions to students’ routines caused by wartime conditions. Given that the total number of university students in Ukraine in 2023–2024 was 1.05 million [29], according to the generally accepted formula, the minimum size of a representative sample with a 3% statistical error and 95% reliability is 1,067 students. While in our survey, the number of students who fully answered all the questions is 1,398. Since the purpose of our study was to analyze the mental condition and academic performance of all students in Ukraine as a separate population group, the sample is representative in this context.

The predominance of female respondents (75%) reflects gender distribution trends in major branches of science in Ukraine’s higher education, particularly in humanities and social sciences, where women constitute the majority. However, there are certain academic programs where men prevail, which can cause additional biases. To address these biases, gender and age were included as variables in the regression model, which indicated that male students demonstrated greater resilience to mental health challenges, showing a 4.0% lower reduction in academic performance. At the same time, while our model accounts for age and gender as influencing factors, a more detailed stratified analysis of variations in academic performance by these variables could provide additional insights. This aspect presents an opportunity for further research to deepen the understanding of how these demographic factors interact with mental health and academic outcomes.

The coefficient of determination of our linear regression is moderate, which demonstrates that our regression can only partially explain the negative changes in students’ academic performance by the symptoms of certain mental health problems. Furthermore, the high value of the regression’s intercept shows the influence of all other factors that were not included in the regression analysis. We believe that this limitation is objective in nature, as students’ academic performance depends on an extremely large number of different variables that cannot be quantified in a single cross-sectional field study. Moreover, the cross-sectional design of our research does not allow us to establish causality, meaning that while our findings demonstrate an association between mental health symptoms and academic performance, they do not confirm a direct cause-and-effect relationship. However, this does not diminish the relevance of our findings, as the T-statistics, F-statistics, and Chi-square test confirm the validity of the model and the significance of the independent variables.

Academic performance is objectively measured by the academic achievements of students, i.e. as the ratio between an output (knowledge that the student has acquired, measured by the grades of the examination or study performance) and input in academic activity measured by the time spent on studying [50]. Unfortunatelly, our cross-sectional study, which included more than 2,300 students from 17 universities from different regions of Ukraine, did not allow us to collect reliable and accurate data on the performance of their study in terms of grades in different courses and knowledge they had acquired. Thus, while our regression model accounts for key mental health-related factors, it does not capture all potential contributors to academic performance, reinforcing the need for further research incorporating additional academic and environmental variables. That is why we used the API variable as a measure of negative changes in academic performance of university students.

### Policy implications

In the third year of full-scale war in Ukraine, mental health remains an urgent issue for the Ukrainian population [51, 52], including students and adolescents. Given that less than 1 million students continue their studies in Ukraine in 2023, there is a significant demand for psychosocial support services for this population group. Additionally, the API among students during the war have long-term negative consequences, as its hinder the acquisition of knowledge and the development of competencies required for future professional careers. Therefore, the mental health problems among students during wartime is gaining a broader socio-economic dimension as it begins to hinder the development of human capital, which is critical for the successful post-war reconstruction of Ukraine’s economy.

The considerable deterioration in the mental health of Ukrainian university students during the war requires a quick response at both the institutional and public policy levels. At the level of Ukrainian higher education institutions, there should be implemented measures for timely monitoring of students’ mental conditions, prevention of mental health disorders, screening for mental health problems, and the implementation of modern interventions. Universities need to recognize the important role of students’ mental health and the need to protect it as part of educational activities, prioritize students’ mental health and turn it into a strategic objective of the higher education institution, apply effective screening methods for the timely recognition of mental health problems among students, introduce modern prevention tools using digital technologies and a system of information materials, and apply a set of interventions (including online) adapted to the peculiarities of the mental condition and needs of Ukrainian students during the war.

At the public policy level, disseminating of best practices for organizing support for students’ mental health during the war, shoud be provided for Ukrainian universities. Furthemore, the government has to secure funding for implementation of relevant programs including: educational activities to promote the concept of mental health, forming basic knowledge about the symptoms of major mental health disorders, introducing screening of students’ mental health at the university level, identifying the first signs of mental health problems, forming risk groups for timely response and determining further diagnostic procedures, as well as implementing adequate psychosocial and therapeutic interventions. Additionally, it is nessasry to increase funding for psychological support cabinets at the universities as well as launching new psychosocial and group support programs based on the best practices of leading universities in the EU, the UK, the US and Canada [53]. Given the well-researched vulnerability of female students to anxiety and depression, as well as their significant representation in certain academic programs, gender-oriented mental health interventions must be integrated into university support systems. These initiatives should go beyond individual counseling to include structured group-based resilience training, crisis adaptation strategies, and tailored psychosocial interventions designed to empower female students in navigating the unique challenges they face during wartime.

## Conclusion

The study demonstrates the high prevalence of mental health problems among Ukranian students during the war as well as the critical role of mental health for academic performance and the normal reproduction of Ukraine’s human capital during the war. In our opinion, only an active public health policy focused on increasing funding and modernizing the national mental health system can ensure active prevention of these threatening phenomena and create the appropriate conditions for the post-war reconstruction of the Ukrainian economy.

## Data Availability

Data is available on request from the last author of the article (Volodymyr Virchenko). It was initially gathered using licensed Qualtrics software and currently stored in Qualtrics certified database at Taras Shevchenko National University of Kyiv.
